# Limited influence of the microbiome on the transcriptional profile of female *Aedes aegypti* mosquitoes

**DOI:** 10.1038/s41598-020-67811-y

**Published:** 2020-07-02

**Authors:** Josephine Hyde, Maria A. Correa, Grant L. Hughes, Blaire Steven, Doug E. Brackney

**Affiliations:** 1 Department of Environmental Sciences, Connecticut Agricultural Experiment Station, New Haven, CT USA; 2 Department of Pathology, Institute for Human Infections and Immunity, Center for Tropical Diseases, Center for Biodefense and Emerging Infectious Disease, University of Texas Medical Branch, Galveston, USA; 3 Center for Vector Biology and Zoonotic Diseases, Connecticut Agricultural Experiment Station, New Haven, CT USA; 4 Present Address: Departments of Vector Biology and Parasitology, Liverpool School of Tropical Medicine, Pembroke Place, Liverpool, UK

**Keywords:** Transcriptomics, Microbiome

## Abstract

The microbiome is an assemblage of microorganisms living in association with a multicellular host. Numerous studies have identified a role for the microbiome in host physiology, development, immunity, and behaviour. The generation of axenic (germ-free) and gnotobiotic model systems has been vital to dissecting the role of the microbiome in host biology. We have previously reported the generation of axenic *Aedes aegypti* mosquitoes, the primary vector of several human pathogenic viruses, including dengue virus and Zika virus. In order to better understand the influence of the microbiome on mosquitoes, we examined the transcriptomes of axenic and conventionally reared *Ae. aegypti* before and after a blood meal. Our results suggest that the microbiome has a much lower effect on the mosquito’s gene expression than previously thought with only 170 genes influenced by the axenic state, while in contrast, blood meal status influenced 809 genes. The pattern of expression influenced by the microbiome is consistent with transient changes similar to infection rather than sweeping physiological changes. While the microbiome does seem to affect some pathways such as immune function and metabolism, our data suggest the microbiome is primarily serving a nutritional role in development with only minor effects in the adult.

## Introduction

There is an increasing recognition that most multicellular organisms harbour microbiota, or a microbiome, that can affect their development, biology, and health. This idea has led to the concept of a “holobiont”, which suggests that the biology of an organism cannot be separated from the mutualistic, commensal, or pathogenic organisms that may stably or transiently exist with that organism^1,2^. Thus, there is an “extended phenotype” for the host that arises from the interactions with its microbiome^3^. Consequently, it can be difficult differentiating the contributions of either the host or its associated microbiome to specific phenotypic outcomes. One of the tools that can be employed to examine the microbiome’s effect on host biology is the availability of microbial free, or axenic hosts^4,5^. Numerous groups have developed axenic systems to study host-microbiome interactions, including *Mus musculus* (mice)^6^, *Sus scrofa* (pigs)^7^, *Caenorhabditis elegans*^8^, fruit fly (*Drosophila melanogaster)*^9^ and *Gallus gallus* (chickens)^10^. By combining these axenic systems with transcriptomics, metabolomics and epigenomics, researchers can now systematically examine the role of the microbiome in host biology^4,11–14^.

As with most complex organisms, mosquitoes possess a taxonomically diverse assemblage of microbes, including bacteria, viruses, fungi and protists^15,16^. While the mosquito microbiome can inhabit multiple tissues within the mosquito, including the germline, malpighian tubules and salivary glands, the majority of microbes reside in the gut^17–19^. Previous studies have found that, compared to vertebrate hosts, the mosquito microbiome is composed of relatively few community members (~ 10–70 Operational Taxonomic Units (OTUs)) which are primarily acquired from the aquatic environment as larvae^16,20–22^. As the aquatic environment has a large influence on the mosquito microbiome, resource use by the mosquitoes at the larval stage is likely to influence the adult core microbiome^23^. The composition of the mosquito microbiome reflects the habitat of the individual with the microbiome varying between sites and individuals from different species at the same location sharing similar microbiomes^24^. Microbiome composition transitions between the larval and adult stages; this shift reflects not only a change in their environment but also a change in their diet, with blood feeding mosquitoes possessing a different core microbiome to their sugar feeding counterparts. Blood meal acquisition results in a decrease in the number of OTUs but an increase in abundance of particular bacterial genera (*Chryseobacterium* and *Delftia* in lab reared *Ae. aegypti*)^24^.

Earlier studies reported that larvae failed to develop under sterile conditions and concluded that the microbiome played an indispensable role during larval development. This was thought to occur through a microbiome-induced hypoxic state, which triggered larval molting and growth^25^. However, the generation of axenic mosquitoes revealed that, with the proper nutritional supplementation, the presence of a microbiome was not essential for development, challenging this notion^26,27^. Transcriptomic analysis of axenic larvae revealed an enrichment of genes associated with energy metabolism similar to axenic *C. elegans* and indicative of dietary restrictions or starvation^28,29^. These findings are consistent with the delayed development time observed while rearing axenic larvae^26^. Together, these data suggest that the microbiome primarily serves a nutritional role during larval development^26,27^. In adult mosquitoes, numerous studies have observed potential effects of the microbiome on pathogen acquisition and transmission^30^, as well as mosquito sensitivity to insecticides^31^. One major example of this is the endosymbiont *Wolbachia* which has been shown to affect viral transmission by mosquitoes, it has also been shown that other members of the microbiome can influence vertical transmission of *Wolbachia*^32^. The capacity of the adult mosquito to transmit infections to humans could be directly influenced by their microbiome at multiple life stages^23^. However, these phenotypes are difficult to verify without an axenic model to systematically test the effects of manipulating the microbiome. Thus, the effects of the microbiome on adult mosquito physiology and behaviour are still largely unknown.

Because mosquitoes represent a sustained and significant public health threat^33^, characterising the precise role of the microbiome on mosquito physiology, development, nutrition, behaviour, and vector competency could aid the development of novel control strategies. The recent development of an axenic mosquito model now allows us to characterise microbiome–host interactions and the effects of removing the native microbiota. In order to address this, we performed transcriptomic analyses on conventionally reared (mosquitoes that possessed a native microbiota) and axenic adult mosquitoes before and after a bloodmeal to look at the effect of the microbiome on host gene expression. We found the total number of differentially expressed genes influenced by the microbiome is more consistent with transient changes akin to infection rather than sweeping physiological changes such as a those associated with bloodmeal.

## Results

### Transcript assembly and differential expression analysis

We used Illumina NextSeq 550 sequencing to transcriptionally profile conventional and axenic adult mosquitoes. Sterility of axenic larvae and adults were tested via culturing of viable bacteria and 16S rRNA gene PCR as previously described^26^. Half of the mosquitoes in each group were provided with a non-infectious bloodmeal and tissues harvested 24 h post blood meal (hpbm). The remaining half were maintained on sugar water. In order to provide more tissue level resolution, midguts and carcasses were processed and analysed separately. Three biological replicates for each treatment group and two tissue sources (midguts and carcasses) resulted in a total of 24 libraries being analysed. Across the libraries, an average of 41.3 million forward and reverse reads (range: 30.0–51.8) per sample were recovered, and this was reduced to an average of 21.2 million paired reads (range: 14.7–25.3) after quality filtering (Table S1). This filtering resulted in a total of 29.3 to 50.6 million quality-filtered reads per treatment, of which an average of 88.49% (range: 86.6–90.7%) mapped to the current assembly of the *Ae. aegypti* genome (AaegL5.0 https://www.vectorbase.org/organisms/aedes-aegypti/lvp_agwg/aaegl5) (Table S1).

**Figure 1:**
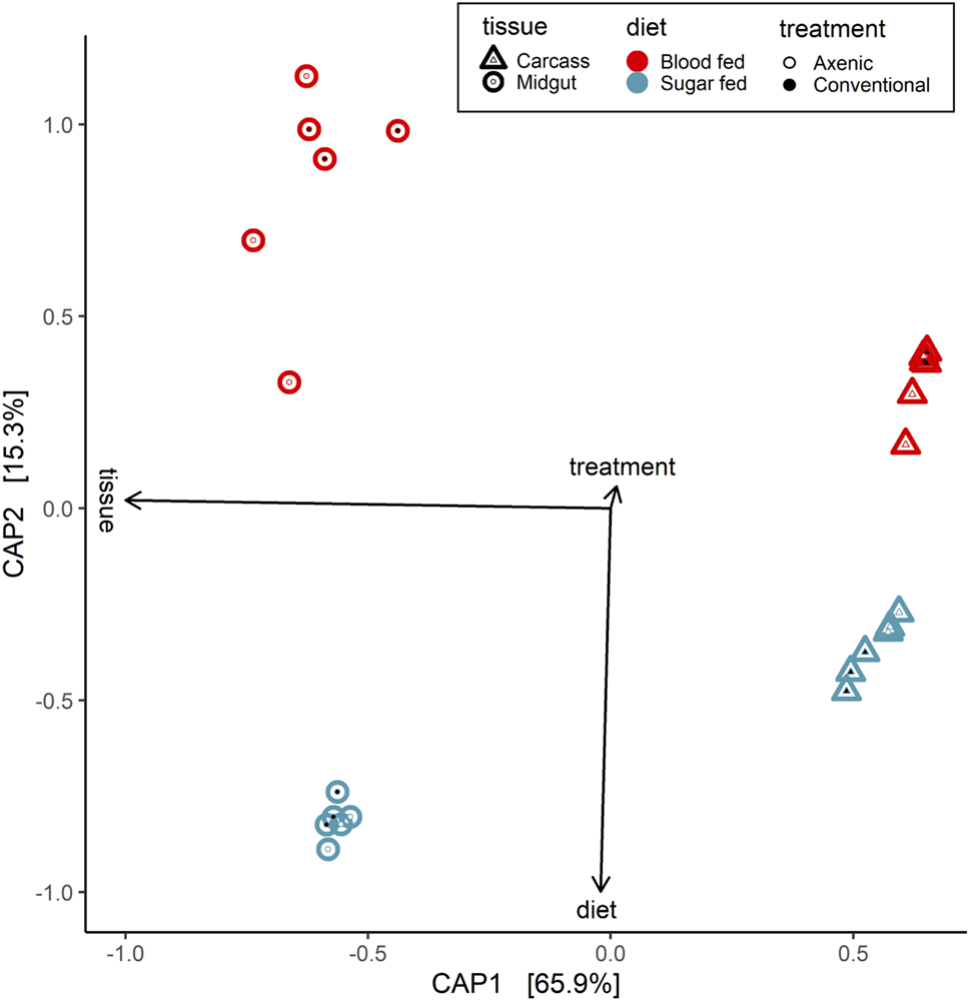
Canonical Analysis of Principal Coordinates (CAP) analysis of transcript abundance by mosquito pool. Constrained Analysis of Principal Coordinates (CAP) ordination for the transcriptomes of the mosquito pools sequenced, based on Jaccard dissimilarity index. CAP axes 1 and 2 explain 65.9 and 15.3% of the variance, respectively. Explanatory variables are indicated by arrows, while diet is indicated by the colours, tissue type by the shape and treatment by the internal dot colour.

Using canonical analysis of principal coordinates (CAP), the data were analysed to identify significant scale differences within the sequence datasets (Fig. 1). It was thought that CAP would confirm that the axenic state would have a significant effect on transcript abundance. However, the first component, which explained 65.9% of the variation in the data (p-value = 0.001), separated the samples by blood-fed status (Fig. 1). The second component, which explained 15.3% of the variation in the data (p-value = 0.001), separated samples by tissue type (Fig. 1). Not only did the microbial status of the mosquito explain much less of the variation between individuals (Fig. 1) but that variation was also not statistically significant (p-value = 0.077). Therefore, due to the low explanatory power in the axenic state, the decision was made to separate the data by individual treatments [midgut blood-fed (MBF), midgut sugar-fed (MSF), carcass blood-fed (CBF), carcass sugar-fed (CSF)] and to make comparison without the confounding factors of diet and tissue type.

We employed two different software packages to identify differentially expressed (DE) transcripts, DESeq2^34^ and Sleuth^35^. Additionally, we applied an effect size threshold (twofold change between conditions) to focus on differences that were most likely to be biologically meaningful. DESeq2 consistently identified more DE transcripts than Sleuth (Table 1). The DESeq2 analyses identified 1,165 DE transcripts whose abundance varied significantly (adjusted p-value < 0.05) in the presence of the microbiome by at least a twofold relative to axenic mosquitoes (Table 1). The Sleuth analyses identified 181 DE transcripts whose abundance varied significantly (qval < 0.05) in the presence of the microbiome by at least a twofold change relative to axenic mosquitoes (Table 1). The overlap in DE transcripts found by both programs only accounted for on average 74% (range: 23.1–97.4%) of the total number of transcripts identified by Sleuth. Thus, Sleuth appeared to be more conservative in identifying DE transcripts and primarily overlapped with the DESeq2 data. Therefore, the Sleuth results were used for further analyses.

**Table 1:** Total transcriptome abundance from both DESeq2 and Sleuth.

	MBF	MSF	CBF	CSF	Total
DESeq2	537	189	261	292	1,165
Sleuth	96	31	39	39	181
Shared	75	30	38	9	152
Unique DESeq2	462	159	223	283	1,013
Unique Sleuth	21	1	1	30	29

### Transcriptional response to the axenic state

**Figure 2:**
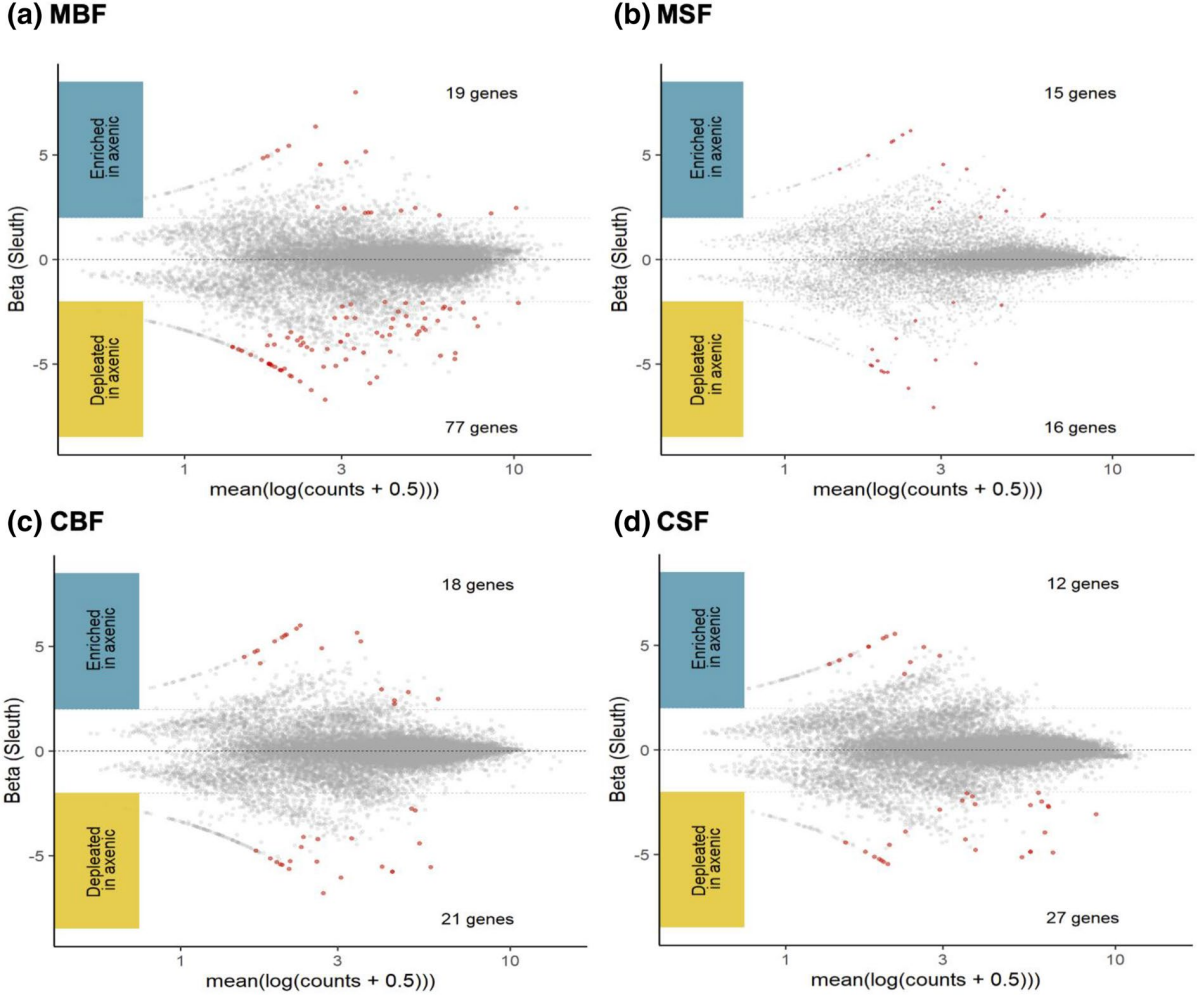
MA plots of axenic versus conventionally reared mosquito genes. The beta value in Sleuth indicates the estimated fold change for each gene. Each dot is representative of one gene; grey dots represent no significant differences between axenic and conventionally raised groups. Red dots represent genes that were either enriched (above the x-axis) or depleted (below the x-axis) and were considered biologically significant in abundance. The four treatment groups investigated were midguts blood-fed (MBF) (**a**), midguts sugar-fed (MSF) (**b**), carcasses blood-fed (CBF) (**c**) and carcasses sugar-fed (CSF) (**d**).

**Figure 3:**
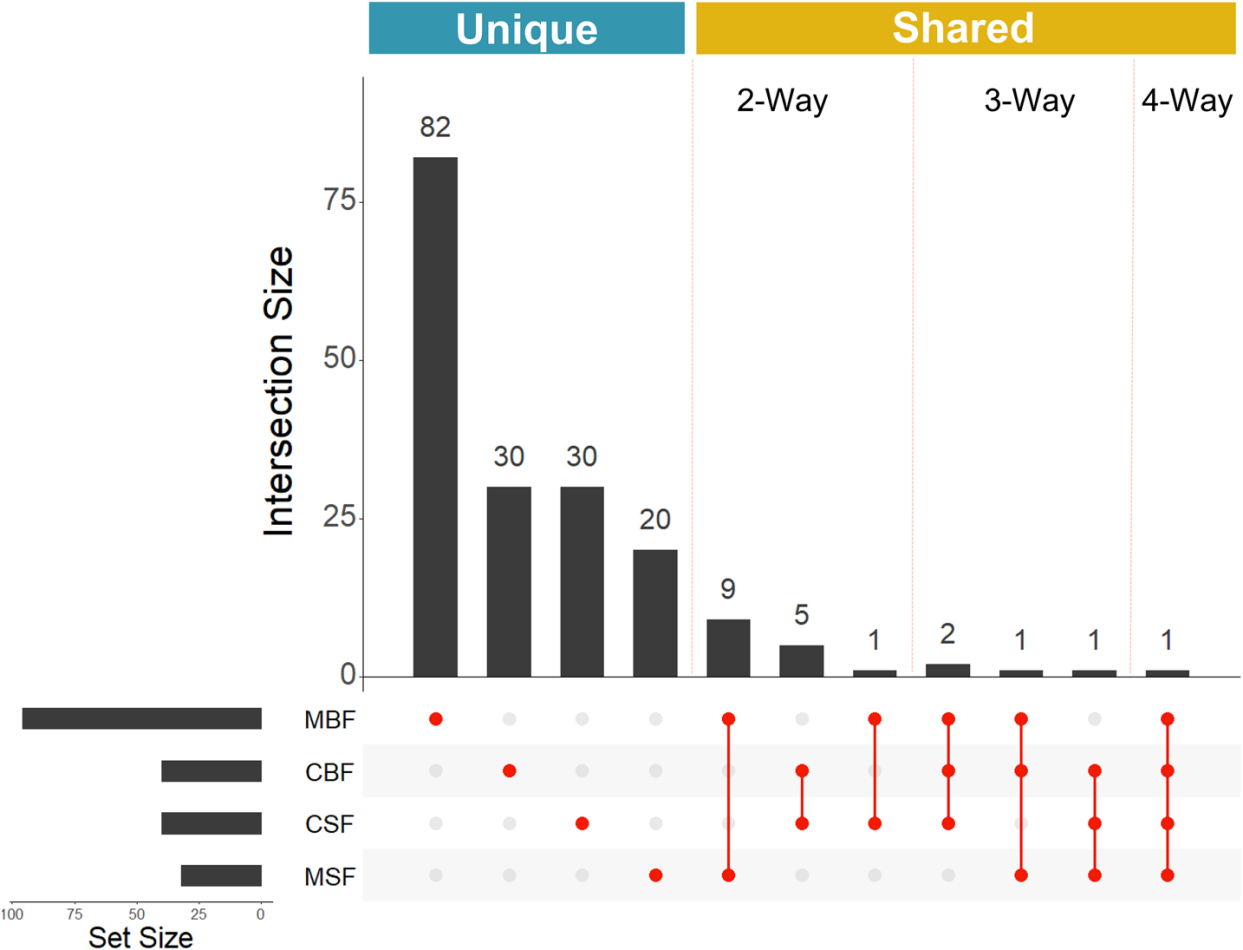
Upset plot of biologically significant genes expressed in the four treatment groups investigated. The plot shows the number of shared genes between the four treatment groups. The set size is the total number of biologically significant genes identified. The interaction size shows how many genes are found either only in one treatment group or in multiple treatment groups. The four treatment groups investigated were midguts blood-fed (MBF), midguts sugar-fed (MSF), carcasses blood-fed (CBF), and carcasses sugar-fed (CSF).

The DE transcripts were visualised by treatment type in MA plots (Bland–Altman plot) (Fig. 2). In all treatment groups, more transcripts were depleted in the axenic state; for the CBF, CSF and MSF treatment groups there was very little difference between the number of DE transcripts either enriched or depleted in the axenic state (Fig. 2). However, there were large differences between the number of enriched and depleted transcripts for the MBF treatment group, with 58 more transcripts showing a decreased abundance in the axenic state. The majority (89.50%) of the DE transcripts were unique to one of the four comparisons (Fig. 3), regardless of whether they were enriched or depleted in the axenic state. Only 15 (8.30%) of the DE transcripts were shared between two comparisons, four (2.21%) of the DE transcripts were shared between three comparisons and only one (0.55%) DE transcript (nesprin-1-like, AAEL025410-RA) was shared between all four comparisons. Additionally, one transcript (UDP-glucuronosyltransferase, AAEL021590 –RA) was enriched in one state (MBF) and depleted in another (MSF) (Fig. 3).

### Annotation of differentially expressed transcripts

**Figure 4:**
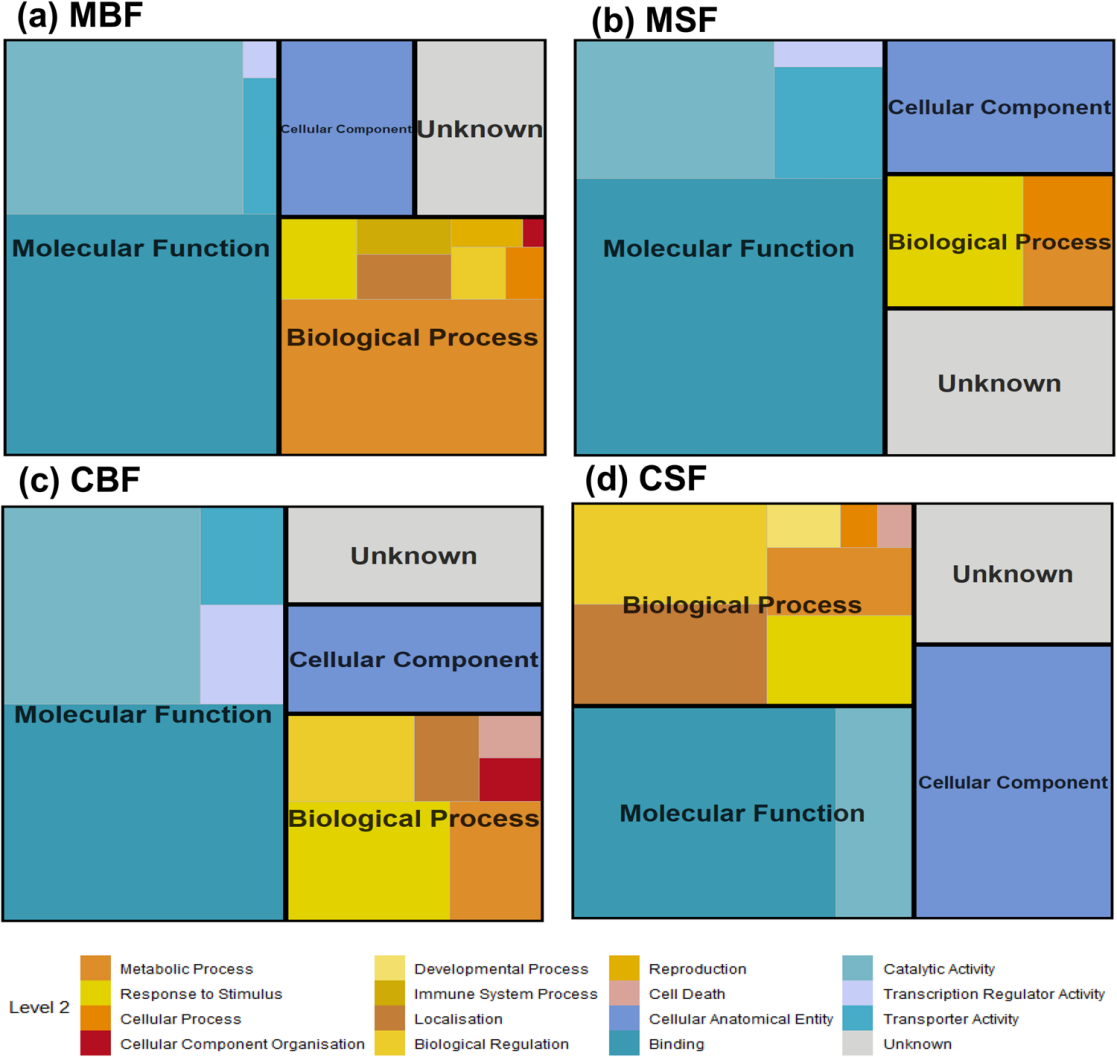
Hierarchical classification of the biologically significant GO terms identified. The treemap shows the comparative abundance of Level 2 GO terms by treatment type, midguts blood-fed (MBF) (**a**), midguts sugar-fed (MSF) (**b**), carcasses blood-fed (CBF) (**c**) and carcasses sugar-fed (CSF) (**d**).

The DE transcripts were annotated using both NCBI and VectorBase. Due to splice variants, 170 genes were identified in the dataset (compared to 181 transcripts), 34 of which were uncharacterised to a functional annotation (Table S2). Using VectorBase^36^, the global classification of gene ontology (GO) terms were extracted for the DE transcripts (Table S2). There were more GO annotations than genes with many genes having multiple GO annotations. However, 47 genes had no known GO annotation. All GO annotations identified could be assigned to one of the three main domains; biological process (30.43%), cellular component (29.89%), and molecular function (39.67%). Analysis of the Level 2 GO annotations revealed that of the 14 identified, only three were unique to a single treatment type (Fig. 4). Developmental process was unique to CSF, and immune system and reproduction processes were both unique to MBF. Four annotations, response to stimulus, metabolic process, binding and catalytic activity were found in all four treatment groups. However, as with the genes, at the deepest level, a large proportion of the GO annotations were unique to each treatment (Fig. S1). However, there were more GO annotations shared between treatments with eight GO annotations shared across all four treatment groups compared to the single gene that was shared between all groups. Splitting the GO annotations into those enriched versus those that were depleted in the axenic state, more GO annotations in total were depleted with only two (protein binding and metal ion binding) being conserved among all four treatments (Fig. S1). Of the transcripts enriched in the axenic state, a single GO annotation (protein binding) was common to the four treatments. Taken together, these observations suggest that there was not a strong conserved biological response to the axenic state that was common to the different treatments.

### Comparison of differential expression in axenic *Aedes aegypti* and *Drosophila melanogaster*

Using orthologues from FlyBase^37^ and VectorBase^36^ DE transcripts from axenic *Ae. aegypti* larvae^29^ and axenic *Drosophila melanogaster*^4^ were compared to those identified in this study. In total, four transcripts identified as differentially expressed due to the axenic state from a study into axenic *D. melanogaster* (Table 2) were also found in this study. All of the genes (defensin-A, defensin-A-like, defensin-C, lysozyme-like) are involved in immune function. Sixteen transcripts identified as differentially expressed due to the axenic state in the larvae were also identified in this study (Table 2). A quarter of the genes shared between adult, and larval axenic mosquitoes were also uncharacterised, and all but two of the remaining genes (cystathionine beta-synthase, phosphoenolpyruvate carboxykinase, purine nucleoside phosphorylase, transferrin, ATP-binding cassette sub-family A member 3, voltage-dependent calcium channel type A subunit 12, phospholipase B1, membrane-associated 1, phospholipase B1, membrane-associated 2, phosphotriesterase-related protein) were involved in nutrient metabolism, catalysis or transport. The remaining two genes were involved in cell death and inflammation (flocculation protein FLO11) toxin receptors (membrane-bound alkaline phosphatase), and wound healing (rho guanine nucleotide exchange factor 11 2).

**Table 2:** Mosquito transcript ID and gene name of transcripts that were shared between axenically raised *Ae. aegypti* and *Drosophila melanogaster.*

Transcript	Name
**Larvae/adult mosquito**	
XM_021848997.1	Membrane-bound alkaline phosphatase
XM_001654437.2	Phospholipase B1, membrane-associated X1
XM_021843337.1	Phospholipase B1, membrane-associated X2
XM_021848596.1	Phosphotriesterase-related protein
XM_011494827.2	Flocculation protein FLO11
XM_021853819.1	Rho guanine nucleotide exchange factor 11 X2
XM_021850833.1	Cystathionine beta-synthase
XM_001647886.2	Phosphoenolpyruvate carboxykinase
XM_001661117.2	Purine nucleoside phosphorylase
XM_001647669.2	Transferrin
XM_021841557.1	ATP-binding cassette sub-family A member 3
XM_021854675.1	Voltage-dependent calcium channel type A subunit X12
XM_001662723.2	Uncharacterised
XM_001662962.2	Uncharacterised
XM_001664030.2	Uncharacterised
XM_021842633.1	Uncharacterised
**Fruit fly/adult mosquito**	
XM_001657243.3	Defensin-A
XM_001657239.3	Defensin-A-like
XM_001657238.3	Defensin-C
XM_021843602.1	Lysozyme-like

### Validation of results

**Figure 5:**
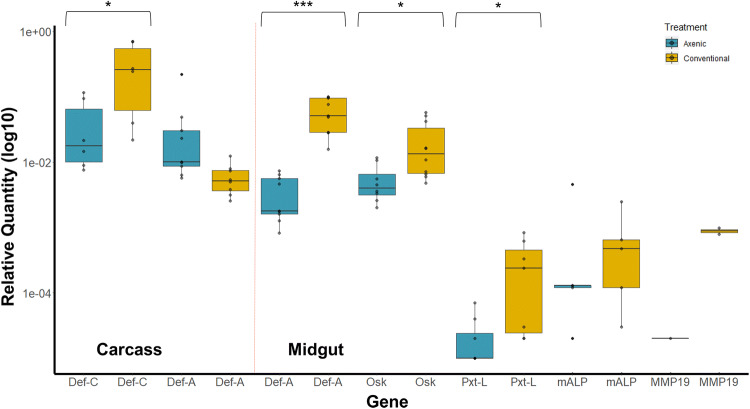
The relative expression of the six genes used to validate transcriptome results. Stars indicate statistical significance; *** < 0.0001 and * < 0.01. Gene codes used are all accepted standard abbreviations and are found in Table 2.

Quantitative PCR (qPCR) was used to validate the RNA-seq results; six genes were chosen based on their abundance profile in the dataset and the ability to design qPCR primers, specifically targeting the relevant transcripts. qPCR was performed on individual mosquitoes rather than pooled mosquitoes, producing a more robust picture of gene expression at an individual level. The genes were only tested on the conditions (i.e. tissue types and blood feed status) that showed significantly different abundance in the sequence-based analysis. Of the seven combinations tested, four comparisons came back statistically significant (Fig. 5). Thus, the qPCR data verifying transcriptional differences due to the lack of microbiome are limited.

**Figure 6:**
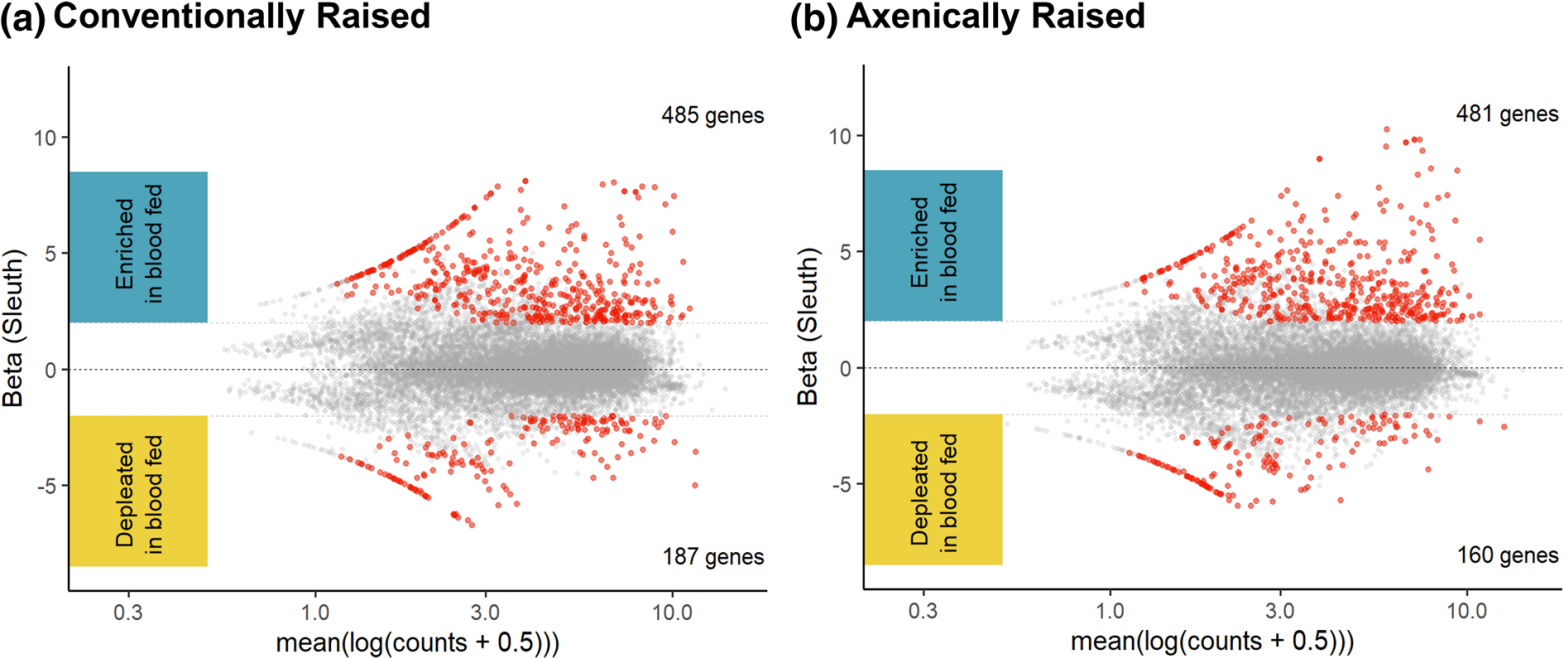
MA plots of significantly expressed mosquito genes during a blood or sugar meal. The beta value in Sleuth indicates the estimated fold change for each gene. Each dot is representative of one gene; grey dots represent no significant differences between axenic and conventionally raised groups. Red dots represent genes that were either enriched (above the x-axis) or depleted (below the x-axis) and were considered biologically significant. The two groups investigated were midguts conventionally raised (**a**), and midguts axenically raised (**b**).

Finally, to confirm that the low number of transcripts discovered was not an artefact of our analysis pipeline, treatment groups were also processed via blood meal status. The two groups were separated by colonisation status or feeding status, and only the midguts were examined. As expected, there was a much larger difference in transcript expression between blood meal status than how the mosquitoes were raised (Fig. 6). There was also a much higher number of DE transcripts with 988 DE transcripts (Fig. 6). The low effect of the axenic gene transcription is further supported in that 54% of the DE transcripts were identical between the axenic and conventionally reared blood-fed groups.

## Discussion

Alterations of transcript abundance identified in association with the axenic state in this study were relatively low, particularly in regard to other transcriptome studies of mosquitoes. For example, microarray studies on whole mosquitoes by Dissanayake et al.^38^ discovered 5,081 DE transcripts between male and female mosquitoes and a further 4,773 DE transcripts between blood-fed and non-blood fed females. Similarly, an Illumina-based deep sequencing study identified 5,969 DE transcripts between blood-fed and non-blood fed mosquitoes; however, a detailed examination shows that a majority of the transcripts identified showed less than a twofold change in expression^39^. Here we only identified 170 DE transcripts due to the axenic state. These numbers are more in line with investigations on the effect of viral and bacterial infections on the host mosquito. Studies on arbovirus infections in *Ae. aegypti* identified 203^40^ and 397^41^ DE genes, and a study on a native *Wolbachia* infection in *Ae. fluviatilis* found 257^42^ DE genes. This suggests that the transcriptional effect of removing the microbiome in adult mosquitoes might be more similar to that of a transient change rather than that of a large physiological change.

An additional explanation for the low recovery of DE transcripts may be methodological. The DESeq2 software package identified a higher number of differentially expressed transcripts (1,165 vs. 181 discovered using Sleuth Table 1). We chose the more conservative analysis as it had the highest congruence between the two methods. It may be that our estimates of DE transcripts are more conservative than in other studies. Additionally, earlier studies used microarray analysis^38,40^ and 454^43^ sequencing, whereas this and more recent studies used Illumina sequencing^39,42,44,45^. Looking at just the RNA Seq studies that used Illumina, including this study, four different versions of the *Ae. aegypti* reference were used, and no two studies employed the same bioinformatics pipeline. Earlier versions of the *Ae. aegypti* genome and less conservative programs to determine differential expression coupled with lower thresholds for determining differential expression might account for some of the differences found in this study. The minimal effect of the axenic state on transcript changes in mosquitoes is supported both by the qPCR and CAP results and the fact that our method still shows large scale changes associated with blood meal acquisition (Fig. 6).

A previous study has documented the transcriptional changes in larval mosquitoes in response to the axenic state and found significantly more DE genes (1,328) than we document here^29^. However, these studies differ in a critical factor. The larvae in the Vogel et al., study could not be reared past the L1 life stage and were only 22 h old. In comparison, we have shown that larvae can be reared in the absence of a microbiome, with the proper nutrition, and there is little detrimental effect on the mosquito^26^. Thus, it is likely that the axenic larvae previously described were in a stressed state due to starvation. This could account for the report of increased transcription of genes involved in amino acid transport, insulin and TOR signalling, fatty acid oxidation, and a decrease in expression of some peptidases. This pattern has widely been identified as a pattern seen in animals undergoing starvation stress, including mosquitoes^29,46–49^.

When trying to identify a core set of genes that were affected by the presence of a microbiome in flies, there were no genes that were identified as being shared between the adult and larval axenic *Ae. aegypti* and *D. melanogaster*, under our selection criteria. This required all genes to have been DE in all three groups with a log abundance of at least twofold (biologically significant) while also being statistically significant. There were several genes; however, that were DE but were not considered biologically significant in all three groups or the orthologue was only found in one of the two species (Table S3). The majority of the genes identified had unknown functions while those shared across species were involved in immune function and within *Ae. aegypti* nutrient metabolism, which supports the idea that the microbiome has a large role in supplying essential nutrients to the mosquito and the microbiome is directly interacting with the host immune system.

Mosquitoes possess a number of molecular and cellular based innate immune mechanisms^50^. Those most commonly associated with defending against bacteria include the Toll, Immune deficiency (Imd) and JAK-STAT signalling pathways which induce the production of antimicrobial peptides^50^. Not surprisingly, evidence suggests that the gut microbiome plays a role in the development and functionality of the mosquito’s innate immune response^51,52^. Anti-microbial peptides are regulated in mosquitoes mainly by the Imd pathway; all of the anti-microbial peptides identified in this study were down-regulated in the axenic state and were all expressed in at least two tissue types. Defensin anti-microbial peptides work against gram-positive bacteria. In this study DEFC (AAEL003832), DEFD (AAEL003857), and two of the three isoforms of DEFA (AAEL003841, AAEL027792) were differentially expressed in the carcass regardless of blood meal status, and DEFA was also differentially expressed in the blood-fed midgut. Another anti-microbial peptide, gambicin (GAM1; AAEL004522), which is regulated by the Toll, Imd, and JAK-STAT signalling pathways in combination, was differentially expressed in the midgut regardless of blood meal status. One immunity-related gene, TLR6 (AAEL023577), was upregulated in the blood-fed midgut. This may be the mosquito attempting to compensate for the decreased expression of the anti-microbial peptides as TLR6 plays a fundamental role in pathogen recognition and innate immune activation.

Vitellogenesis (yolk deposition) is vital for successful reproduction in mosquitoes. The process is regulated by the juvenile hormone (JH) pathway^53–55^. Previous studies have shown that nutritionally deprived mosquito larvae have lower levels of JH resulting in the production of fewer eggs as adults^39^. The influence of the gut microbiome on nutritional status has been observed in multiple studies^56,57^. For example, there is a significant delay in the developmental time of axenically reared *Ae. aegypti* compared to their conventionally reared counterparts^26,58^. In this study, we found an enrichment of juvenile hormone epoxide hydrolase (AAEL011313) in blood-fed midguts of axenic adults. This enzyme is a negative regulator of JH and its enrichment may indicate an active effort on behalf of the axenic mosquito to conserve nutritional resources potentially resulting in lower egg production. Similarly, we observed a reduction in forkhead A2 (AAEL003173) transcripts in the axenic blood-fed midgut. Forkhead box (FOX) proteins are transcription factors that regulate the expression of genes associated with cell growth, proliferation and differentiation^51^. In fact, suppression of fat body associated FOX family proteins have been shown to reduce egg production in *Ae. aegypti*; however, midgut associated FOX family members have not been assessed for their role in oogenesis^59^. While the axenic mosquitoes tested in Correa et al.^26^ did not show a statistical difference in the number of eggs laid, there are still potentially lifelong effects from an axenic state during development. Experiments using gnotobiotic *Ae. aegypti* and *Aedes atropalpus* mosquitoes demonstrated that multiple different bacterial species supported oogenesis in *Ae. aegypti* while only one bacterial species supported normal egg production in *Ae. atropalpus*^60^. When investigated the differences between treatments within *Ae. atropalpus* nutrient levels of stored lipids appeared to make the difference in egg production^60^. Additionally, a study examining the role of the gut microbiome and blood digestion demonstrated that bacteria plays a key role in the digestion of proteins and that the lack of bacteria could deprive the mosquito of essential nutrients and affect oocyte maturation in *Ae. aegypti*^61^.

In other axenic species, there has been evidence of intestinal remodelling, with the presence of a microbiome required for normal development^4,5,62,63^. In both vertebrates and invertebrates studied, the absences of a gut microbiome affected gut morphology in a number of ways, including epithelial cell renewal, cell composition and spacing, and a decrease in total intestinal size^4,64^. Several genes were upregulated in the midgut that could relate to intestinal remodelling and function including genes involved in muscle formation (AAEL018288), mucus production (AAEL019619, AAEL023490), microtubule formation (AAEL019567), and ciliary formation (AAEL018303). Additionally, genes that are involved in the regulation of intestinal homeostasis (AAEL014566, AAEL007658) were downregulated in the axenic state, which might suggest that it is more difficult for axenic mosquitoes to recover from insults.

In summary, while this transcriptional analysis has identified several differentially expressed genes that could point to specific gene expression or metabolic pathways that may be remodelled in the absence of a microbiome, the overall effect of the axenic state on the adult mosquito is relatively small. This raises interesting questions as to how mosquitoes acquire and regulate their internal bacterial load to avoid the potential negative consequences of unchecked bacterial growth and potential phenotypic outcomes associated with altered microbiome compositions, such as vector competence.

## Materials and methods

### *Aedes aegypti* rearing

Sterilisation of the *Aedes aegypti* eggs was carried out as previously described^26,65^. Briefly *Ae. aegypti* eggs were collected from colony mosquitoes, and in a sterile hood the eggs were serially rinsed for five minutes in 70% ethanol, followed by a five-minute wash in a 3% bleach and 0.2% ROCCAL-D (Pfizer) solution, and then again for five minutes in fresh 70% ethanol. The sterilised eggs were then rinsed three times in autoclaved DI water and placed in a Petri dish filled with phosphate-buffered saline (PBS). Eggs were hatched in a vacuum oven (Precision Scientific Model 29) at 25 Hz for 15 min at room temperature. The sterility of larvae and mosquitoes was tested via culturing viable bacteria and 16S rRNA gene PCR as previously described^26^. Conventionally reared mosquitoes were established by colonising axenic mosquitoes with bacteria from a homogenized colony mosquito.

Larvae were transferred from the Petri dish to individual wells of six-well tissue culture plates; each well contained 5 ml of sterilised DI water and a 0.6 g plug of liver yeast agar^18^. After pupation, the mosquitoes were transferred to autoclaved mosquito emergence chambers. All the mosquitoes were fed on filter sterilised 10% sucrose solution. Approximately a week after the first emergence, half the mosquitoes were blood-fed using a circulating water bath and membrane feeder. Mosquitoes were fed sterile defibrinated sheep blood (Hemostat Laboratories), and axenic Swiss Webster mouse pelts (provided by Dr Andrew Goodman, Yale University) were used as the membrane in lieu of parafilm. All feeds were carried out in a biosafety cabinet under sterile conditions.

### RNA extractions and library preparation

Midguts were dissected from sugar-fed and 24 hpbm groups in sterile PBS and pools of five midguts or carcasses/group were placed in mirVana lysis buffer. There were three pooled replicates of each group. RNA was extracted using the mirVana miRNA Isolation kit according to the manufacturer’s instructions (Ambion). Total RNA was quantified using the BR RNA assay on the Qubit (Thermo Fisher Scientific) and quality assessed using an RNA 6,000 chip on an Agilent 2,100 Bioanalyzer (Agilent Technologies). Library construction and sequencing were performed at the Next Generation Sequencing Core at the University of Texas Medical Branch. 1 µg of total RNA was used to construct libraries using the NEBNext Ultra II RNA Library kit according to the manufacturer’s recommendations (New England Biolabs). The resultant DNA libraries with nonhomologous 5′ and 3′ ends were analysed by qPCR to determine the template concentration of each library. The 24 samples were pooled and sequenced on an Illumina NextSeq550 using a high-output flow-cell and paired-end 75 base reads following the manufacturer's protocol.

### Data analysis

Sequences were quality checked using FastQC^66^ and quality filtered using Trimmomatic 0.36^67^, sequencing adapters and reads with Phred-equivalent scores of < 33 were removed at this stage. Potential sequence contamination arising from phiX, human, and sheep (blood source) were removed using BBDuk 36.62 and BBMap 36.62^68^. Quality filtered reads then pseudoaligned to the *Aedes aegypti* genome (assembly AaegL5) using Kallisto 0.43.1^69^. Data were then imported into RStudio and analysed with DESeq2^34^, and Sleuth^35^ to determine differential abundance. Default parameters were used in both analyses.

Gene Ontology terms (GO) were obtained from VectorBase annotations. Statistical analyses were performed in R using the packages agricolae^70^ and vegan^71^.

### Real-time quantitative PCR assays

Quantitative PCR (qPCR) assays verified patterns in the transcriptome datasets. Mosquitoes were raised, dissected, and subjected to RNA extraction as described above The quantitative PCR assays were performed using the iTaq Universal SYBR Green One-Step Kit (Bio-Rad, Hercules, California, USA) in a 20-µl reaction volume, 50 ng of RNA was added to all reactions, and all samples were tested in duplicate and NTC, and NRT controls were included. The genes were chosen if they did not have any splice variants, and primers could be designed to cover exon-exon boundaries. The primer sequences used in these assays are listed in Table S4.

## Supplementary information

Supplementary file1 (PDF 13 kb)

Supplementary file2 (XLSX 305 kb)

Supplementary file3 (PDF 96 kb)

## Data Availability

Sequences and related information determined in this work can be found at GenBank accession #'s SRR11209355-SRR11209378 and Bioproject PRJNA609359.
