# Supplementary figures and images for: Limited influence of the microbiome on the transcriptional profile of female Aedes aegypti mosquitoes
Source: Sci Rep. 2020 Jul 2;10:10880. doi: 10.1038/s41598-020-67811-y (PMC7331810; doi:10.1038/s41598-020-67811-y)

(a) **Enriched**

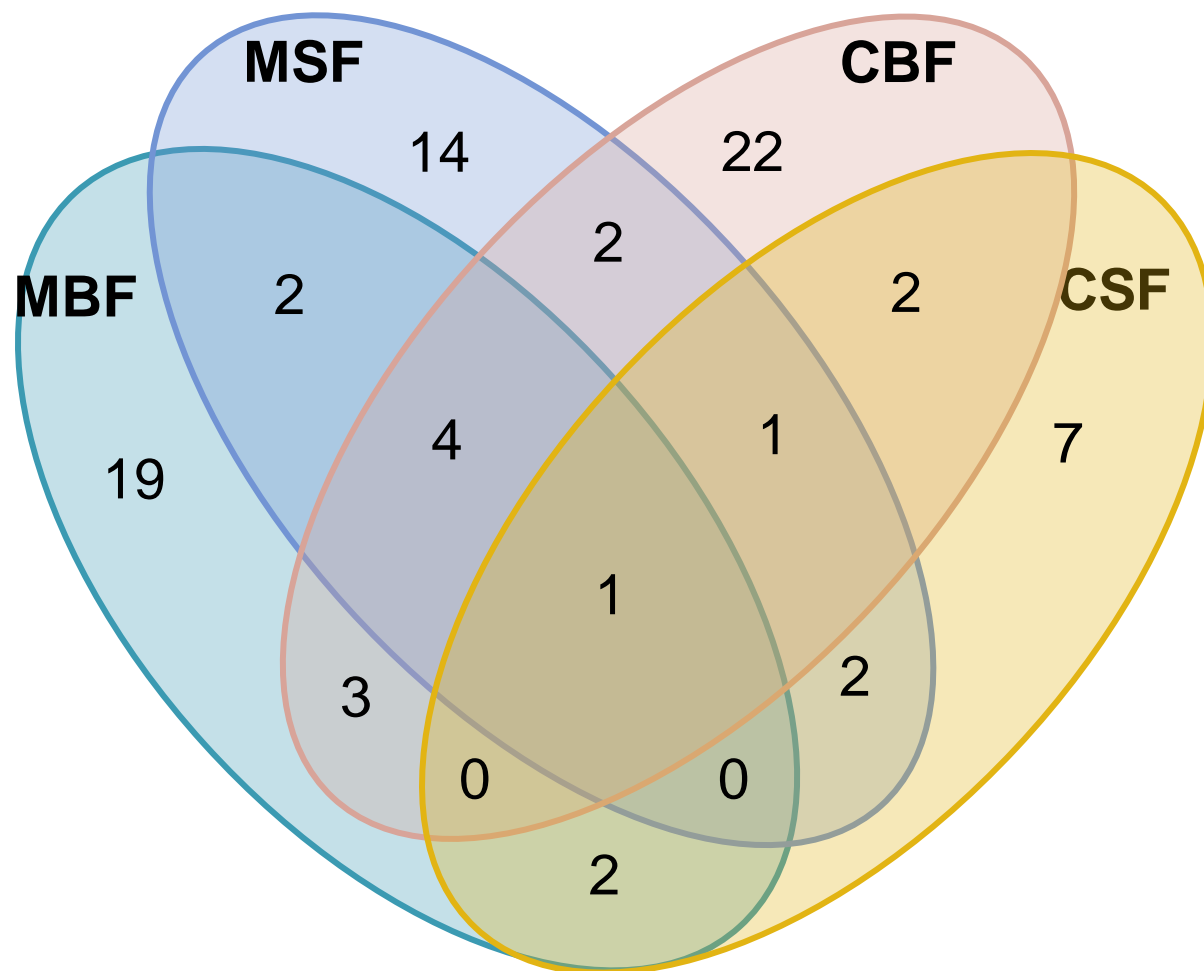

(b) **Depleted**

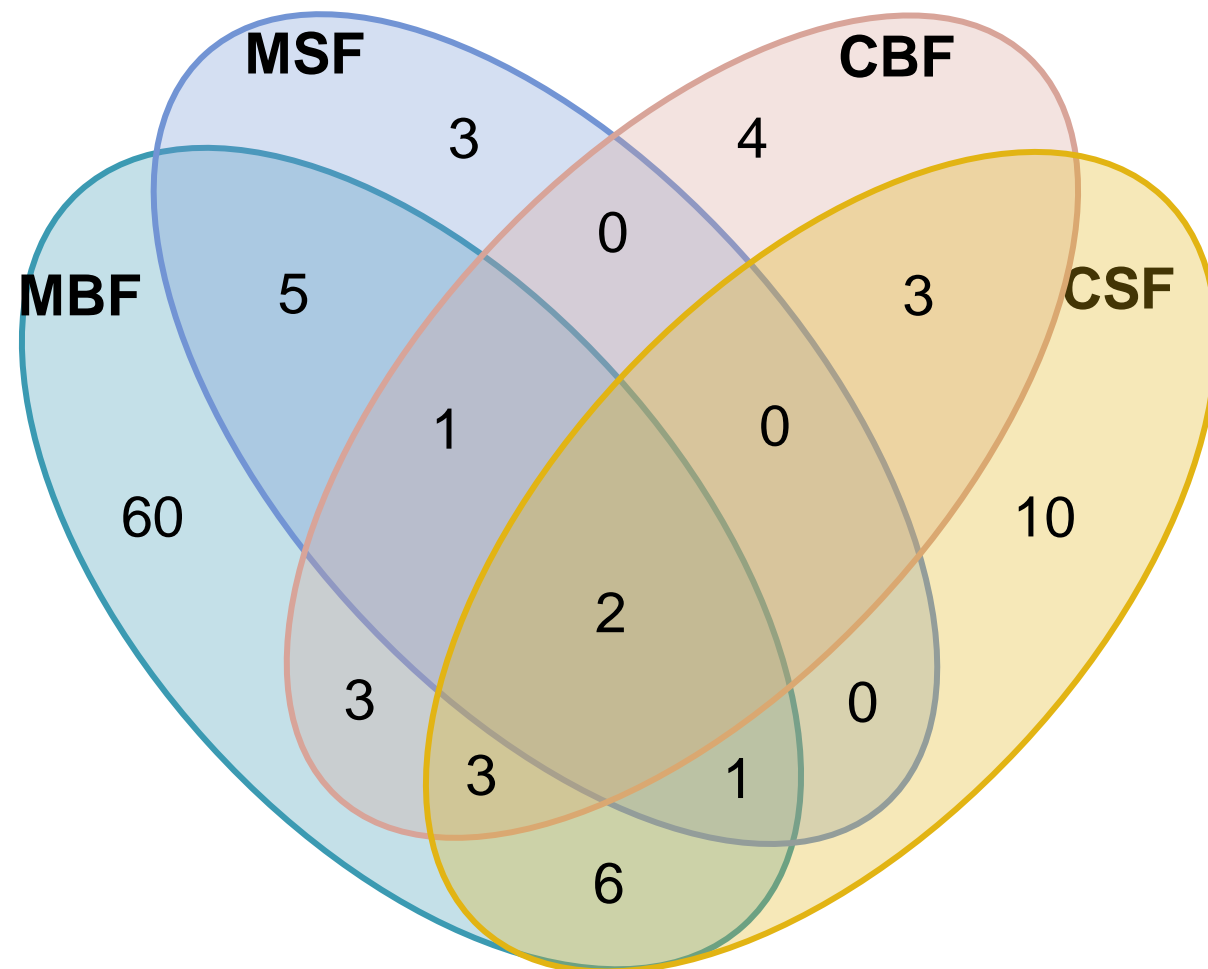

Supplement: Supplementary file 1 — Supplementary file1 (PDF 13 kb) [file 41598_2020_67811_MOESM1_ESM.pdf]
