# Supplementary material for: Limited influence of the microbiome on the transcriptional profile of female Aedes aegypti mosquitoes
Source: Sci Rep. 2020 Jul 2;10:10880. doi: 10.1038/s41598-020-67811-y (PMC7331810; doi:10.1038/s41598-020-67811-y)
Supplement: Supplementary file 3 — Supplementary file3 (PDF 96 kb) [file 41598_2020_67811_MOESM3_ESM.pdf]

Table S4: Complete list of primers designer for qPCR used in this study

Target ID	NCBI	Forward Primer	Reverse Primer	Product Length	Position	Abbreviation	P. adju
XM_001656365.2	Maternal Effect Protein Oskar	GACGACTTCTTCCTTTCGCT	CGTTAATGTAGGGGGCCTTC	145	ex3/ex4	Osk	0.04
XM_001662006.2	Matrix Metalloproteinase-19	CGCAACCTGAGGATATAGAGGCAA	CGGGTTGTGGTAAGTGGCAG	91	ex1/ex2	MMP19	0.20
XM_021848998.1	Membrane-Bound Alkaline Phosphatase	GAGCGGTTGATTTGGTGCGT	CATCACTTCCTCGGCTCGGATA	114	ex1/ex2	mALP	0.32
XM_021856546.1	Defensin C	GGACCAACCATGAAGTCGATCA	CCGGCAGTTCATCGAAAAGAGAG	139	ex2/ex3	Def-A	0.0001 (M), 0.09 (C)
XM_001657238.3	Defensin A	TAGCAACTTCACCGTTCGCA	TCATCAAACAAAGAGTTGGCGTAAG	197	ex1/ex2	Def-C	0.03
XM_001648979.3	Chorion Peroxidase-Like	CCGGAAGAAGACGATGGGTG	GAGCGTTTCTGGTGATGGCT	176	ex1/ex2	Pxt-L	0.03
